# Surface Association of Flaxseed Oil on Cassava Starch Granules via Prolonged Mild Thermal Treatment: Structural, Pasting, Textural, and Emulsifying Properties

**DOI:** 10.3390/foods15122099

**Published:** 2026-06-11

**Authors:** Wendou Xue, Zehong Liang, Zhaodi Lu, Chunli Wang, Yang Liu, Shunxin Zhang, Xinwei Wang, Hongxin Jiang

**Affiliations:** College of Food Science and Technology, Henan University of Technology, Zhengzhou 450001, China; 17549218610@163.com (W.X.); lzh1753558680@163.com (Z.L.); 15138606334@163.com (Z.L.); 18137851468@163.com (C.W.); 13526558654@163.com (Y.L.); zhangsx202509@163.com (S.Z.)

**Keywords:** cassava starch, mild thermal treatment, oil-modified, surface association, pasting properties, emulsifying properties

## Abstract

The objective of this study was to evaluate the effect of prolonged mild thermal treatment (65 °C, 7 days) in the presence of flaxseed oil (0.16%, *w*/*w*), on the structural, pasting, texture, and emulsifying properties of cassava starch (CS). The resulting sample was designated as CS-oil-h. Confocal laser scanning micrographs showed oil on the interface of starch granules promoted granule agglomeration. DSC and FTIR analyses showed no detectable evidence of amylose–lipid complexes or new covalent bonds. Compared to CS, CS-oil-h exhibited slight variations in pasting temperature and peak time, and significantly lower peak, trough, breakdown, final, and setback viscosities. CS-oil-h gel showed higher hardness, adhesiveness, gumminess, and chewiness when compared to the CS gel. Crucially, CS-oil-h exhibited the best emulsifying ability (60.8%, volume of emulsion layer relative to total tube volume after 30 min standing) and emulsion stability (94.1%, after 7 days of storage). The result suggested that the prolonged mild thermal treatment may have promoted putative surface association between flaxseed oil and the surface of starch granules, which resulted in inhibition of pasting viscosity and improved gel properties and emulsifying ability.

## 1. Introduction

Cassava starch exhibits high viscosity, high transparency, a bland taste, low pasting temperature, and relatively slow retrogradation, making it preferable for food and non-food applications [1,2,3]. However, native cassava starch also has low thermal resistance and shear stability and lacks hydrophobic properties [4,5,6,7]. Therefore, chemical, enzymatic, and physical modifications are employed to improve functional performance of cassava starch, thereby broadening its applications [8,9,10,11].

Starch–lipid complexes, formed through interactions between starch and lipids, can alter starch pasting, thermal, rheological, textural, and digestion properties [12,13,14,15]. Oil-modified starch, prepared by high-temperature heating starch with trace edible oil, shows reduced digestibility, improved shear stability, and enhanced emulsification ability compared to its native counterpart [15,16,17]. These characteristics make oil-modified starch promising for use in products such as low glycemic index foods, dairy products requiring viscosifying agents with high shear and thermal stability, and processed meats [15]. It has been reported that oil-modified starch can improve the gel strength and water-holding capacity of surimi gels [18]. In meat applications, oil-modified starch serves as an effective animal fat replacer, improving gelation, water retention, and microstructure, while significantly reducing total fat and saturated fatty acid content [19]. Moreover, oil-modified starch combines the gel-enhancing ability of starch with the lubricating taste of oil to provide a fat-mimicking texture [16].

According to previous studies, oil-modified starch was successfully prepared by mixing cross-linked/acetylated starch with oil added in amounts ranging from 0.2% to 2% and heat-treated at temperatures ranging from 90 to 140 °C for 2–6 h [14,16,17,18,19]. Although high-temperature oil modification (≥90 °C) has been widely applied to crosslinked or acetylated starches, such conditions have been reported to impair the short-range ordered structure and helical structure of starch granules and may compromise the intrinsic properties of granular starch. Furthermore, most published studies on oil-modified starch have used chemically pre-modified substrates (cross-linked or acetylated starch) rather than native starch, leaving the behavior of native cassava starch under mild oil-treatment conditions largely unexplored. By contrast, whether trace quantities of flaxseed oil under prolonged mild thermal treatment below the gelatinization temperature can alter the surface properties and interfacial behavior of native cassava starch without forming amylose–lipid complexes remains unresolved.

To overcome the limitation, mild thermal treatment with low dosage of oil was used to prepare oil-modified starch. The treatment temperature (65 °C), which is above the glass transition temperature of amorphous starch at ~13% moisture content, was selected to facilitate oil–starch surface interactions while preserving granular integrity [20]. The treatment duration of 7 days and oil dosage of 0.16% (*w*/*w*, relative to dry starch) were determined based on preliminary experiments. Preliminary results showed that the emulsifying performance gradually increased with treatment duration and approached a plateau after 7 days, with no substantial additional improvement observed at longer treatment times. In this study, flaxseed oil was used to modify cassava starch at 65 °C for 7 days, and the effects of mild thermal treatment and flaxseed oil on its structure and properties were systematically investigated. Particularly, changes in powder flowability, pasting behavior, texture, thermal characteristics, and emulsification ability were analyzed. It is hypothesized that the combined mild thermal treatment and low dosage of oil can significantly improve the pasting, textural, and emulsifying properties of cassava starch compared with either treatment alone. This work aimed to provide new insights into starch–oil interactions and expand the application potential of oil-modified starches in the food industry.

## 2. Materials and Methods

### 2.1. Materials and Chemicals

Cassava starch was obtained from Zhejiang Henxin Biotechnology Co., Ltd. (Jinhua, China). The starch contained 12.94% moisture, while the protein, ash, and apparent amylose contents were 0.10%, 0.21%, and 25.1% on a dry basis, respectively. Apparent amylose content was determined using the iodine colorimetric method. Flaxseed oil was purchased from Fuzhou Xingfu Foods Co., Ltd. (Fuzhou, China). The flaxseed oil used in this study mainly consists of triglycerides rich in polyunsaturated fatty acids, predominantly α-linolenic acid (39–60%), followed by linoleic acid (~15–18%) and oleic acid (~13–19%), together with minor amounts of saturated fatty acids [21]. All chemicals and reagents used in this study were of analytical grade.

### 2.2. Preparation of Oil-Modified Starch

The moisture content of cassava starch (300 g, dry basis) was adjusted to 30.0% by adding 118.53 mL of distilled water. Flaxseed oil (0.48 g, 0.16%, *w*/*w*, based on dry starch weight) was mechanically dispersed in 33 mL of distilled water by continuous stirring immediately before spraying onto the starch, bringing the total moisture content of the starch–oil–water mixture to 35.0%. The mixture was then homogenized in a grinder (FT-800A, Fangtai Electric Appliance, Dongguan, China) at 3000 rpm for 10 cycles (1 min per cycle) with intermittent pauses between cycles to minimize sample heating. The prepared sample was spread as a thin layer on trays and dried in a forced-air oven at 40 °C until the moisture content reached approximately 13.0%. CS-oil and native cassava starch were then heated at 65 °C for 7 days, and named CS-oil-h and CS-h, respectively.

### 2.3. Confocal Laser Scanning Microscopy (CLSM)

Granule morphology of starch was analyzed using CLSM (LSM 900, Zeiss, Jena, Germany) following a method reported by Zhao et al. [17]. Briefly, 1 mL of 1% starch suspension was stained with 20 μL fluorescein isothiocyanate (FITC) stock solution (20 mg/10 mL in methanol) and 10 μL Nile Red stock solution (10 mg/5 mL in ethanol). After incubation at 4 °C for 12 h, the samples were washed with deionized water at least three times to remove unbound dyes, then put on the slide and covered with a cover slip, finally observed with CLSM at 488 nm and 560 nm excitation with 40× objective lens. Representative images from at least three randomly selected fields were collected under identical imaging conditions.

### 2.4. Thermal Properties

Thermal properties of starch were determined using a differential scanning calorimeter (DSC 6000, PerkinElmer, Shelton, CT, USA), following the method described by Wang et al. [22] with minor modifications. Briefly, the sample (10.0 mg, dry basis) was mixed with water at a ratio of 1:3 (*w*/*w*), sealed in an aluminum pan, and equilibrated at 4 °C overnight. The prepared samples were heated from 10 °C to 160 °C at 10 °C/min, using an empty aluminum pan as a reference. The DSC enthalpy values were normalized on a dry starch basis.

### 2.5. Fourier Transform Infrared Spectroscopy (FTIR)

Fourier transform infrared spectra of starch were analyzed using a Fourier transform infrared spectrometer (Nicolet 6700, Thermo Fisher Scientific, Waltham, MA, USA) following a method reported by Zhao et al. [14]. Samples were prepared using the KBr pellet method. Spectra were recorded over a wavenumber range of 4000 to 400 cm^−1^ at a resolution of 4 cm^−1^, accumulating 64 scans per spectrum. Spectra were baseline-corrected and normalized to the band at 1022 cm^−1^. No mathematical deconvolution was performed.

### 2.6. Powder Flowability

The angle of repose of starch was determined using the method described by Buzera et al. [23]. A funnel (cone angle 60°, orifice inner diameter 10 mm) was fixed onto the test tube rack, with the bottom of the funnel neck positioned 6 cm above the tabletop. Starch (15.0 g) was weighed and poured into the funnel, allowing it to fall freely and form a cone. The height (H) and base diameter (L) of the powder cone were then measured, and the angle of repose (α) was calculated using the following formula. All samples were re-equilibrated to a uniform moisture level prior to testing.

(1) $$\alpha =\tan^{-1}\frac{2H}{L}\;$$

### 2.7. Pasting Properties

Pasting properties of starch were analyzed using a rapid viscosity analyzer (RVA 4800, Perten Instruments, Hagersten, Sweden) following a method reported by Zhang et al. [24]. Each starch suspension (8.0%, *w*/*w*, dry basis, based on total dry sample mass) with total weight of 28 g was equilibrated at 50 °C for 1 min, heated to 95 °C at 6 °C/min, held at 95 °C for 5 min, and then cooled down to 50 °C at 6 °C/min. The paddle speed was maintained at 160 rpm throughout the analysis except for the first 10 s at 960 rpm. At the end of the procedure, the starch paste was collected for subsequent analysis.

### 2.8. Textural Properties

Textural properties of the starch pastes prepared in Section 2.7 were determined using a texture analyzer (TA-XT Plus, Stable Micro Systems, Surrey, UK) equipped with a cylindrical probe (P/0.5R, 12.7 mm in diameter and 40 mm in height) according to the method reported by Gu et al. [25]. The starch paste from the RVA was transferred into a sealed container (20 mm in diameter × 40 mm in height), stored at 4 °C for 24 h, and then equilibrated at room temperature (25 °C) for 1 h before texture profile analysis. Pre-test speed of 1.0 mm/s, test speed of 2.0 mm/s, post-test speed of 10 mm/s, and trigger force of 5 g were set for the determination. The depth of penetration of the probe into the starch paste during the test was 15 mm, and the time required was 7.5 s. Two compression cycles were applied with a 5 s interval between cycles. Three independently prepared gels were analyzed per sample.

### 2.9. Transmittance

Transmittance of starch paste was measured according to the method reported by Zhang et al. [26] with minor modifications. Starch (1%, *w*/*w*) was cooked at 95 °C for 15 min. Distilled water was used as a blank control. After cooking, the starch paste was sealed and allowed to cool at room temperature (25 °C) for 1 h. The cooled paste was gently stirred to minimize sedimentation and immediately transferred into cuvettes (1 cm path length) for transmittance measurement at 640 nm using a spectrophotometer (UV-2550, Shimadzu, Kyoto, Japan).

### 2.10. Emulsifying Ability and Emulsion Stability

The emulsion was prepared according to the method reported by Song et al. [27] with slight modifications. An ungelatinized starch suspension (8%, *w*/*w*, dry starch basis, and 100 mL) was first prepared in a glass beaker, and then mixed with 50 mL soybean oil (oil-to-water volume ratio 1:2). The oil–water mixture was homogenized using a high-speed homogenizer (FJ300-SH, Biaomo, Shanghai, China) equipped with a 28 mm probe at 12,000 r/min for 2.0 min at room temperature in a 250 mL tall-form glass beaker. Immediately after homogenization, 25 mL of the emulsion was transferred into graduated test tube. The emulsions were stored in graduated 25 mL test tubes at room temperature, and changes in emulsion volume were recorded at regular intervals. The emulsifying ability (EA) was calculated by dividing the volume of the emulsion layer by the total volume of the tube (25 mL) after 30 min of standing.

(2) $$\mathrm{EA}\;(\%)={\;V}_{0}/25\;\times \;100$$

where V_0_ is the volume of the emulsion layer in the tube. After storing the emulsion for 7 days at room temperature, the volume of the emulsion layer and the total volume of the tube were recorded to calculate the emulsifying ability (EA*). The emulsion stability (ES) was calculated as follows:

(3) $$\mathrm{ES}\;(\%)\;=\;{\mathrm{EA}}^{*}/\mathrm{EA}\;\times \;100$$

### 2.11. Statistical Analysis

Unless otherwise stated, all experiments were performed in triplicate, with data expressed as mean ± standard deviation. Data visualization was conducted using Origin software (2022 Version, OriginLab, Northampton, MA, USA). Statistical significance (*p* < 0.05) between groups was determined using analysis of variance (ANOVA) followed by Duncan’s multiple range test using the SPSS 24.0 statistical software (IBM SPSS Inc., Chicago, IL, USA).

## 3. Results

### 3.1. Granule Morphology

**Figure 1:**
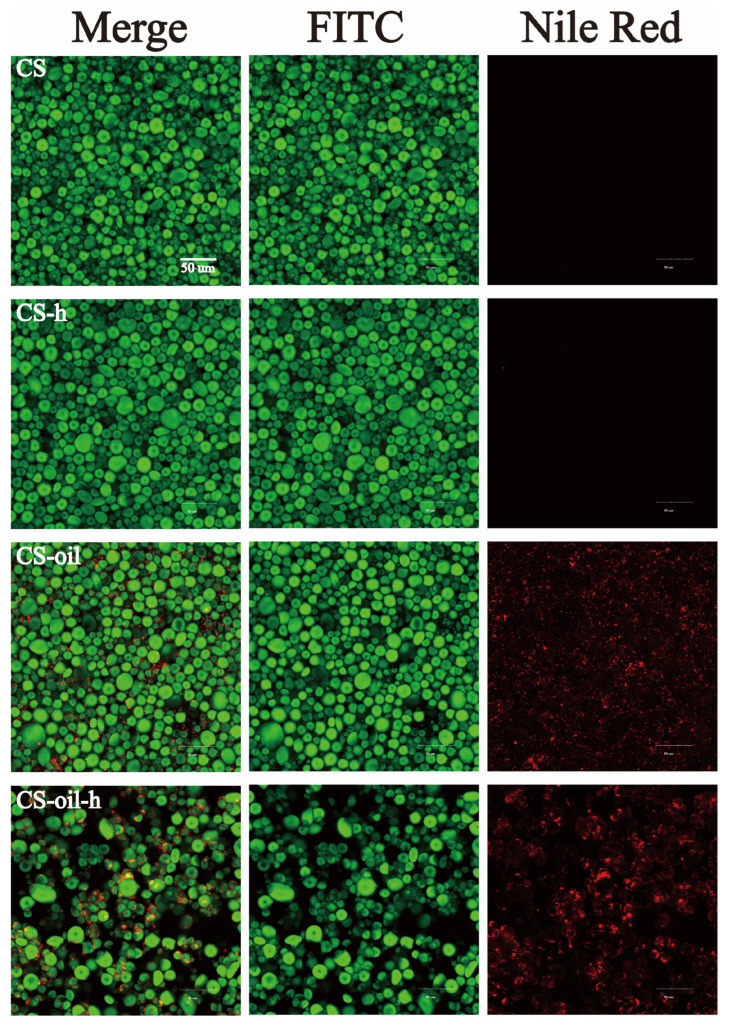
CLSM images of cassava starch before and after heat treatment, with and without flaxseed oil. CS: native cassava starch, oil: flaxseed oil, and h: mild thermal treatment at 65 °C for 7 days.

CLSM was used to investigate the location of flaxseed oil in the CS granules. The images are presented in Figure 1, where the starch and oil phases are shown in green and red, respectively. As shown in Figure 1, CS granules exhibited spherical, oval, or truncated spheres shapes [2]. CS and CS-h showed no detectable red fluorescence signal in the Nile Red channel, confirming the absence of oil in these samples. In CS-oil, discrete punctate red-fluorescent signals were predominantly distributed throughout the interstitial spaces between starch granules, while only minor signals were observed at the granule periphery, suggesting that oil and starch existed largely as separate phases with limited surface association [28]. In contrast, CS-oil-h showed red fluorescence signals markedly concentrated at the periphery of starch granules, with an apparent overlap with the green FITC channel in the merged image, accompanied by a more agglomerated granule distribution. These results suggested that the prolonged mild thermal treatment may have promoted the surface association between flaxseed oil and starch granule surface. The phenomenon of oil adhesion to starch surfaces is consistent with the finding reported by Zhao et al. [14].

### 3.2. Thermal Properties

**Figure 2:**
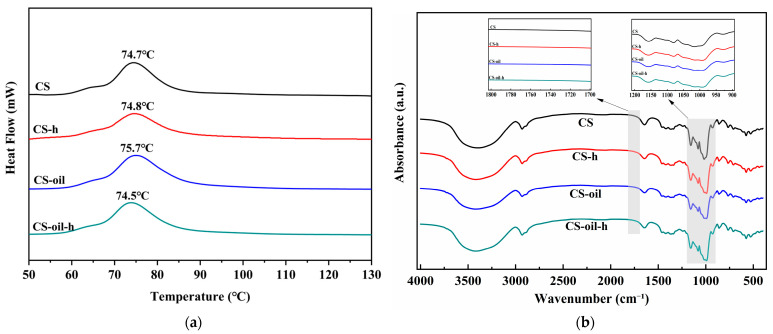
(**a**) DSC thermograms and (**b**) FTIR spectra of cassava starch before and after heat treatment, with and without flaxseed oil. CS: native cassava starch, oil: flaxseed oil, and h: mild thermal treatment at 65 °C for 7 days.

The DSC thermograms of starches are shown in Figure 2a, and their gelatinization temperatures and enthalpy changes are summarized in Table 1. Figure 2a presents the 50–130 °C region, which encompasses all thermal transitions relevant to starch gelatinization and potential starch–lipid interactions. Slight variations were observed in gelatinization temperatures among the starches (Table 1), indicating that mild thermal treatment had little effect on them. As seen in Figure 2a, no detectable DSC endotherm attributable to starch–lipid complexes was observed above 90 °C in starches treated with flaxseed oil. The result is consistent with the finding reported by Zhao et al. [14]. This is because flaxseed oil contains a large proportion of polyunsaturated fatty acids, which makes it more difficult to form a complex with amylose [21,29]. In addition, the amount of oil added (0.16%) was low, and the thermal treatment temperature (65 °C) might not be sufficient to promote the formation of amylose–lipid complex [30,31].

The gelatinization temperature range (ΔT = T_c_ − T_o_) reflects the uniformity of the crystalline structures within starch granules. CS-h showed a slightly increased ΔT relative to CS, implying that mild thermal treatment alone induced limited rearrangement within starch granules. This aligns with previous findings that heat treatment slightly disrupts the uniformity of starch crystalline structure and results in slight variation in ΔT [30,32]. Compared with CS, CS-oil showed a significantly increased ΔT value (20.3 °C), indicating that flaxseed oil addition broadened the gelatinization transition range and increased the structural heterogeneity of starch granules. This may be related to the non-uniform distribution of oil around starch granules observed in CLSM images (Figure 1), which could interfere with water penetration during gelatinization. In contrast, CS-oil-h exhibited a slightly reduced ΔT compared with CS-oil, suggesting that prolonged mild thermal treatment partially reorganized the starch–oil system.

Enthalpy change (ΔH) of starch is the energy required to melt the double-helical crystalline structure. The ΔHs of CS-h and CS-oil-h were significantly lower than those of CS and CS-oil (Table 1), respectively, suggesting that mild thermal treatment slightly disrupted the double-helical crystalline structure. This finding is consistent with previous observations [33]. In addition, the ΔH of CS-oil (10.0 J/g) was higher than that of CS (8.1 J/g), indicating that the addition of flaxseed oil altered the gelatinization behavior of starch. This increase may be attributed to the presence of oil around starch granules, which could hinder water penetration and reduce the uniformity of granule hydration during gelatinization, thereby increasing the energy required to disrupt the double-helical crystalline structure. The ΔH of the CS-oil-h was about 8.8 J/g, which was between CS-h and CS-oil. Similar findings were reported by Lan et al. [34]. The higher ΔH of CS-oil may be attributed to restricted water penetration during gelatinization caused by the presence of oil around starch granules, whereas prolonged mild thermal treatment may partially disrupt ordered double-helical structures, resulting in the lower ΔH observed in CS-oil-h.

### 3.3. FTIR Spectra

The FTIR spectra of different starch samples in the range of 4000–400 cm^−1^ are presented in Figure 2b. Compared with CS, the absence of new peaks in the spectra of CS-oil and CS-oil-h suggested that no major new covalent bonds were detected between oil and starch under the experimental conditions. In addition, no detectable absorption at 1730–1750 cm^−1^ was observed in CS-oil or CS-oil-h, which may be attributable to the low oil content (0.16%, *w*/*w*) falling below the instrument detection limit rather than the definitive absence of ester carbonyls. The C-O band at 1047 cm^−1^ remained a single unsplit peak in all samples, suggesting that no detectable V-type starch–lipid complexes were formed. Similar results were also reported for oil-modified crosslinked starch prepared at 140 °C with an oil addition level of 0.4% [16]. This may be attributed to the relatively large molecular size and steric hindrance of triglycerides, which restrict their incorporation into the amylose helical cavity. Therefore, the oil may be associated with the granule surface through surface physical association without detectable evidence of chemical modification under the methods used, consistent with CLSM micrographs (Figure 1).

### 3.4. Powder Flowability

**Figure 3:**
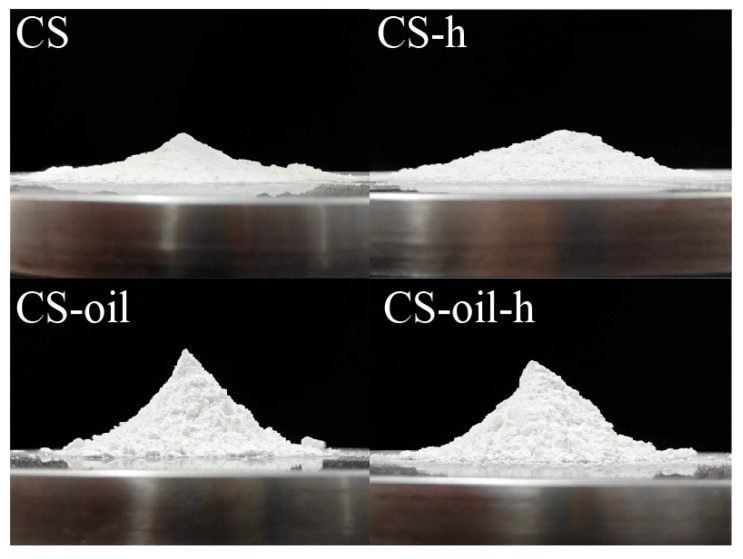
Images of angle of repose of cassava starch before and after heat treatment, with and without flaxseed oil. CS: native cassava starch, oil: flaxseed oil, and h: mild thermal treatment at 65 °C for 7 days.

The angle of repose refers to the angle between the surface of a powder pile and the horizontal plane when the powder is at rest [35]. It is used to evaluate the powder flowability, and a smaller inclination angle reflects lower friction and better flowability [36]. The angles of repose data are summarized in Table 1, and images of the powder cones are presented in Figure 3. As shown in Table 1, the addition of flaxseed oil largely increased the angle of repose in CS-oil. This suggested that the oil dispersed around the starch granules (Figure 1), thus enhancing surface friction and adhesion [37].

The angle of repose of cassava starch increased slightly after prolonged mild thermal treatment, both with and without flaxseed oil (Table 1 and Figure 3). This indicated that the mild thermal treatment caused roughening of the granule surface and increased friction [38]. The highest angle of repose (46.2°) was observed in CS-oil-h, although no significant difference was found compared with CS-oil, indicating that oil addition rather than mild thermal treatment was the dominant factor affecting powder flowability.

### 3.5. Pasting Properties

**Figure 4:**
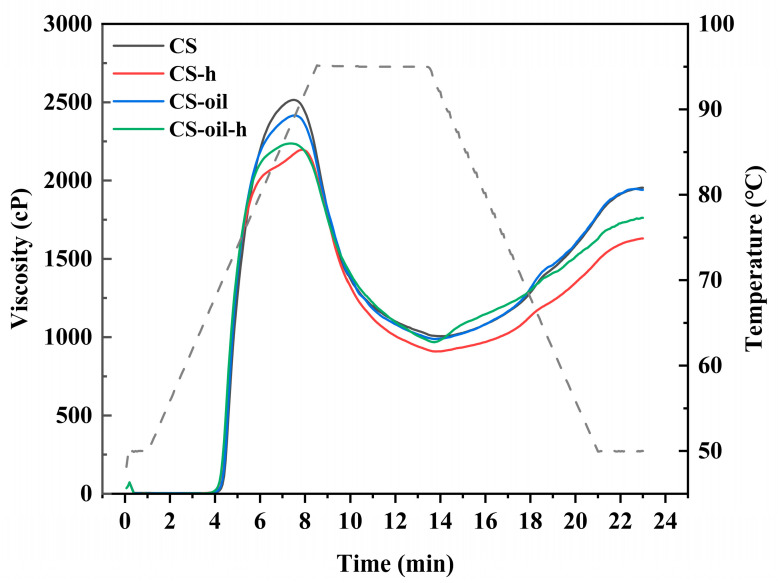
Pasting curves of cassava starch before and after heat treatment, with and without flaxseed oil. CS: native cassava starch, oil: flaxseed oil, and h: mild thermal treatment at 65 °C for 7 days.

Starch pasting refers to the irreversible process in which starch granules in excess water absorb water and undergo structural changes due to gelatinization under heating condition, resulting in formation of a viscous paste or gel [39]. Peak time, pasting temperature, peak viscosity, trough viscosity, final viscosity, breakdown viscosity, and setback viscosity are crucial indicators for starch-pasting behavior [40]. The pasting properties of cassava starch before and after mild thermal treatment, with and without flaxseed oil, are shown in Figure 4, and data are summarized in Table 2. Slight variations in pasting temperature and peak time were observed among the starches (Table 2), indicating that mild thermal treatment of cassava starch with oil had limited effect on them.

The introduction of flaxseed oil into cassava starch slightly reduced the peak viscosity (Figure 4 and Table 2), suggesting that the flaxseed oil adhering to the starch granule surfaces inhibited the maximum swelling of the granules [41]. After mild thermal treatment, the peak, trough, breakdown, final, and setback viscosities of CS-h and CS-oil-h significantly decreased, compared to those of their respective non-thermal-treated counterparts (Table 2), indicating that mild thermal treatment had significant effect on them. This is because the prolonged mild thermal treatment leads to structural densification of starch granules [42,43], which thereby reduces the maximum swelling of starch granules. Notably, the PV of CS-oil-h (2237 cP) remained slightly higher than that of CS-h (2174 cP), and similar trends were observed for FV and SB (Table 2). This may be because the oil distributed around starch granules acted as a physical barrier, partially limiting heat- and moisture-induced structural rearrangement within granules during thermal treatment, thereby partially retaining the paste viscosity and reassociation ability of starch. These results suggested that flaxseed oil and mild thermal treatment influenced starch pasting behavior through different mechanisms, and their combined effect was not simply additive. The findings suggested that the pasting properties of cassava starch could be modified by mild thermal treatment of starch with flaxseed oil.

### 3.6. Textural Properties

Table 3 shows the hardness, adhesiveness, gumminess, and chewiness of CS viscoelastic gel before and after mild thermal treatment, with and without flaxseed oil. It is noteworthy that all treated samples (CS-h, CS-oil, and CS-oil-h) exhibited lower pasting viscosities but higher gel hardness than CS, suggesting that different structural mechanisms may dominate during heating and cooling processes [44]. During heating, both surface-associated oil and heat-induced structural densification may restrict starch granule swelling, thereby reducing paste viscosity. During cooling, however, the reduced granule disintegration and enhanced structural integrity may facilitate the formation of a denser gel network through increased intermolecular association, resulting in higher gel hardness. Mild thermal treatment significantly increased the hardness, adhesiveness, gumminess, and chewiness of cassava starch viscoelastic gels, both with and without flaxseed oil, indicating that heat treatment was a primary contributor to texture enhancement. This may be because the prolonged mild thermal treatment increased starch granule rigidity, which enhances the rigidity of the swollen granule and molecular interactions [45,46]. Compared to native cassava starch, CS-oil increased the hardness, gumminess and chewiness, suggesting that surface-associated flaxseed oil may have moderately strengthened the gel structure during cooling. CS-oil-h exhibited the highest gumminess (103) and adhesiveness (196 g·s). This may be attributed to the combined contributions of heat-induced increases in granule rigidity together with possible changes in granule surface characteristics associated with surface-associated oil, both of which may have contributed to the increased adhesiveness of the starch viscoelastic gel [47,48].

### 3.7. Transmittance

Transmittance of starch paste is an important indicator for applications [49]. The results are shown in Table 1. The transmittance values of CS and CS-h were 11.9% and 11.2%, slightly higher than those of CS-oil and CS-oil-h (10.5% and 9.9%, respectively), indicating mild thermal treatment and the addition of flaxseed oil reduced the transmittance of the starch paste. The lower transmittance may be attributed to increased light scattering caused by surface-associated oil, granule aggregates, and restricted granule swelling during gelatinization [50]. The lowest transmittance (9.9%) was observed in CS-oil-h, suggesting that the combined flaxseed oil and mild thermal treatment further enhanced light scattering within the starch paste system.

### 3.8. Emulsifying Properties

**Figure 5:**
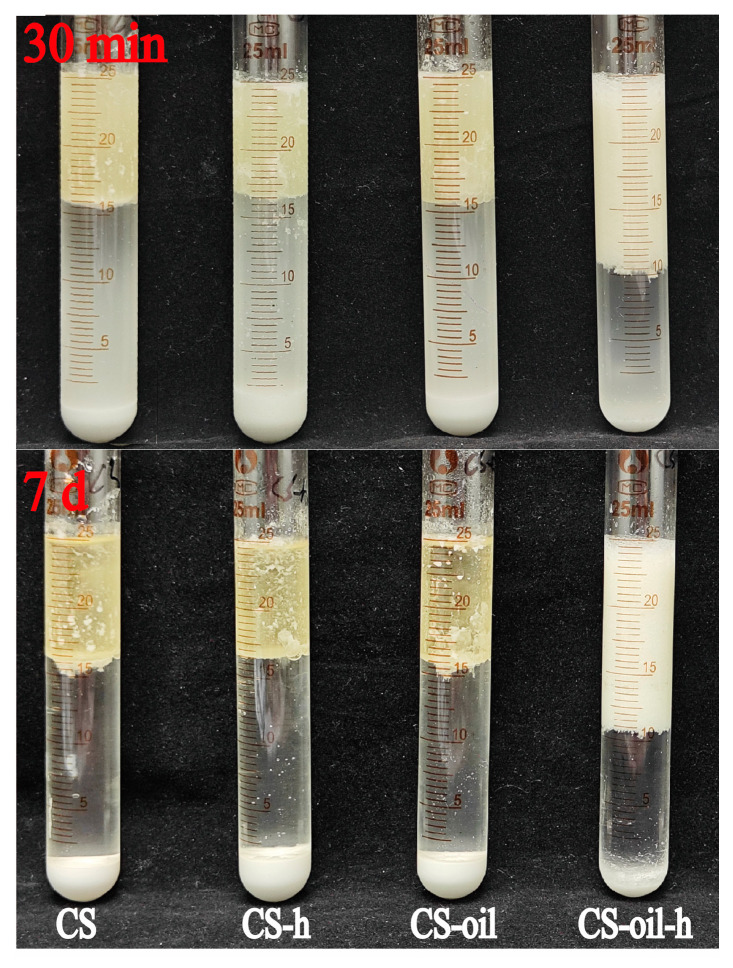
Images of emulsifying ability of cassava starch before and after heat treatment, with and without flaxseed oil. 7 d: seven days. CS: native cassava starch, oil: flaxseed oil, and h: mild thermal treatment at 65 °C for 7 days.

Emulsifying properties of starch refer to the ability to facilitate the formation and retention of oil–water dispersed systems, as evaluated by the volumetric method [51]. Images of emulsion are shown in Figure 5. CS failed to form an emulsion, and the oil–water mixture underwent phase separation within 30 min. This result indicated that native cassava starch had no emulsifying ability. This finding aligns with a previous report [52]. Neither mild thermal treatment nor the addition of flaxseed oil alone enabled the cassava starch to form an emulsion. In contrast, CS-oil-h successfully formed a visibly stable emulsion layer (Figure 5), with EA of 60.8%. Moreover, after 7 days of storage, the CS-oil-h emulsion maintained high emulsion stability (94.1%, Figure 5), indicating that improved emulsion volume-retention behavior compared with the other samples. This aligns with previous findings that high-temperature treatment of starch with oil increases the ES [16,53]. The enhanced emulsifying properties of CS-oil-h may be related to changes in the granule surface characteristics induced by the combined mild thermal treatment and oil association. It is hypothesized that surface triglycerides may have partially increased the hydrophobic character of the granule surface, thereby improving the interaction between starch particles and the oil–water interface.

### 3.9. Proposed Surface Association of Oil and Starch by Mild Thermal Treatment

**Figure 6:**
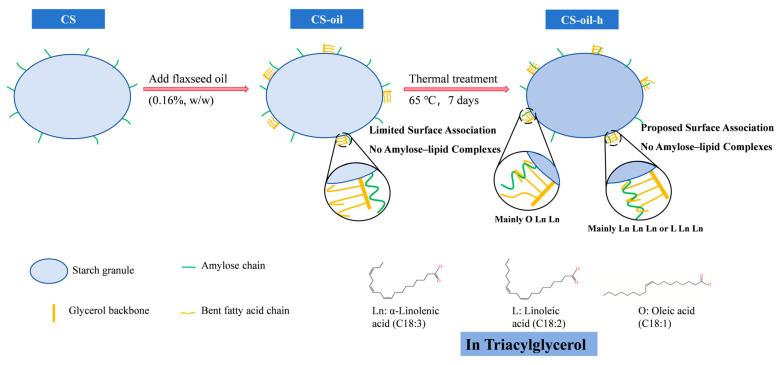
Proposed model for surface association between flaxseed oil and cassava starch during prolonged mild thermal treatment. CS: native cassava starch, oil: flaxseed oil, and h: mild thermal treatment at 65 °C for 7 days. Ln, L, and O represent α-linolenic acid, linoleic acid, and oleic acid residues within triacylglycerol molecules, respectively.

Figure 6 illustrates the proposed surface association behavior between cassava starch and flaxseed oil during the prolonged mild thermal treatment. Under prolonged mild thermal treatment at 65 °C for 7 days, flaxseed oil appeared to be enriched on the surface of starch granules, as evidenced by CLSM images (Figure 1). However, lipid molecules did not show detectable evidence of entering the helical cavity to form typical V-type starch–lipid inclusion complexes, which was evidenced by the absence of characteristic endothermic peaks above 90 °C in DSC thermograms and the lack of new absorption bands in FTIR spectra (Figure 2, respectively). These results suggest that the interaction between flaxseed oil and cassava starch was more likely dominated by surface association rather than inclusion complex formation or chemical modification. It is hypothesized that prolonged mild thermal treatment promoted molecular rearrangement at the granule surface, thereby facilitating closer association between starch chain segments and surface-associated oil. The polyunsaturated fatty acids chains in flaxseed oil, characterized by cis-double-bond-induced bent molecular conformations, may have contributed to localized physical interlocking or transient surface association with accessible starch chain segments at the starch granule surface [54,55,56]. However, this remains a proposed model rather than a directly demonstrated molecular mechanism. The surface-associated oil is further proposed to have partially restricted water penetration into starch granules during gelatinization, thereby limiting granule swelling and contributing to the reduced pasting viscosity observed in RVA analysis. During cooling, the relatively intact granule structure and enhanced granule–granule interactions may have facilitated formation of a denser gel network, resulting in increased gel hardness. In addition, the altered surface characteristics of CS-oil-h may have improved its interaction with the oil–water interface, contributing to the enhanced emulsion volume-retention behavior observed in Figure 5. Nevertheless, direct evidence regarding surface hydrophobicity, interfacial adsorption behavior, and molecular-level starch–oil interactions was not obtained in the present study and requires further investigation.

## 4. Conclusions

The prolonged mild thermal treatment of CS (65 °C, 7 days) with flaxseed oil showed no detectable evidence of amylose-lipid complex formation or formation of new covalent bonds, but it may have enhanced the physical surface association between the surface of CS granules and the oil. This proposed surface association, combined with the structural changes in CS during thermal treatment, modified the starch properties. These changes included reductions in pasting viscosity, paste transmittance and powder flowability, increases in gel hardness, adhesiveness, gumminess, and chewiness, and improvements in emulsifying ability and stability. The oil-modified starch prepared in this study showed potential for applications in food systems requiring modified emulsifying and textural properties. Future studies should further explore alternative or assisted processing strategies to shorten treatment duration while maintaining comparable functional properties, as well as evaluate the performance of this starch in specific food matrices and larger-scale processing conditions and conduct molecular-level and interfacial characterization to further verify the proposed surface-association mechanism.

Several limitations of this study should be acknowledged. First, only a single oil type, dosage, thermal treatment condition, and one prepared batch were evaluated; therefore, the generalizability and reproducibility of these findings require further verification using independently prepared batches under broader processing conditions. Second, some control conditions, including water-sprayed controls with matched pretreatment history, were not included in the present study. Third, the oxidative stability of flaxseed oil during the 7-day treatment at 65 °C was not monitored, and surface characteristics of the modified starch were not directly quantified.

## Figures and Tables

**Table 1:** Thermal properties, powder flowability, and paste transmittance of cassava starch before and after heat treatment, with and without flaxseed oil ^1, 2, 3^.

Samples	Thermal Properties					Powder Flowability (Angle of Repose, °)	Paste Transmittance (%)
	T_o_ (°C)	T_p_ (°C)	T_c_ (°C)	ΔT (°C)	ΔH (J/g)		
CS	67.0 ± 0.3 ^a^	75.0 ± 0.2 ^ab^	85.4 ± 0.4 ^b^	18.4 ± 0.1 ^c^	8.1 ± 0.2 ^c^	18.3 ± 0.7 ^c^	11.9 ± 0.1 ^a^
CS-h	66.4 ± 0.2 ^ab^	74.8 ± 0.3 ^ab^	85.6 ± 0.2 ^b^	19.2 ± 0.4 ^b^	7.1 ± 0.0 ^d^	22.7 ± 1.3 ^b^	11.2 ± 0.0 ^b^
CS-oil	66.3 ± 0.5 ^ab^	75.3 ± 0.7 ^a^	86.6 ± 0.1 ^a^	20.3 ± 0.4 ^a^	10.0 ± 0.5 ^a^	45.2 ± 0.3 ^a^	10.5 ± 0.1 ^c^
CS-oil-h	65.8 ± 0.2 ^b^	74.5 ± 0.2 ^b^	84.7 ± 0.2 ^c^	19.0 ± 0.1 ^bc^	8.8 ± 0.3 ^b^	46.2 ± 0.0 ^a^	9.9 ± 0.0 ^d^

^1^ Data are presented as mean ± standard deviation, based on three independent analyses. Within a column, means bearing different superscript letters indicate a significant difference at *p* < 0.05. ^2^ CS: native cassava starch, oil: flaxseed oil, and h: mild thermal treatment at 65 °C for 7 days. ^3^ T_o_, T_p_, and T_c_: onset, peak, and conclusion gelatinization temperatures, respectively, ΔT: gelatinization range (T_c_ − T_o_), and ΔH: enthalpy change.

**Table 2:** Pasting properties of cassava starch before and after heat treatment, with and without flaxseed oil ^1, 2, 3^.

Samples	PT (°C)	Peak Time (min)	PV (cP)	TV (cP)	BD (cP)	FV (cP)	SB (cP)
CS	69.55 ± 0.00 ^a^	7.5 ± 0.0 ^c^	2515 ± 7 ^a^	1006 ± 6 ^a^	1510 ± 1 ^a^	1955 ± 13 ^a^	949 ± 6 ^a^
CS-h	68.75 ± 0.00 ^c^	7.8 ± 0.0 ^a^	2174 ± 8 ^d^	901 ± 9 ^d^	1273 ± 5 ^c^	1629 ± 5 ^c^	728 ± 18 ^c^
CS-oil	69.17 ± 0.03 ^b^	7.6 ± 0.0 ^b^	2452 ± 16 ^b^	988 ± 1 ^b^	1451 ± 5 ^b^	1951 ± 14 ^a^	961 ± 10 ^a^
CS-oil-h	68.55 ± 0.08 ^d^	7.3 ± 0.0 ^d^	2237 ± 16 ^c^	968 ± 3 ^c^	1269 ± 28 ^c^	1762 ± 14 ^b^	794 ± 27 ^b^

^1^ Data are presented as mean ± standard deviation, based on three independent analyses. Within a column, means bearing different superscript letters indicate a significant difference at *p* < 0.05. ^2^ CS: native cassava starch, oil: flaxseed oil, and h: mild thermal treatment at 65 °C for 7 days. ^3^ PT: pasting temperature, PV: peak viscosity, TV: trough viscosity, BD: breakdown viscosity, FV: final viscosity, and SB: setback viscosity.

**Table 3:** Textural properties of cassava starch before and after heat treatment, with and without addition of flaxseed oil ^1, 2, 3^.

Samples	Hardness (g)	Adhesiveness (g·s) ^4^	Gumminess	Chewiness
CS	98 ± 2 ^c^	135 ± 1 ^d^	60 ± 1 ^d^	57 ± 2 ^d^
CS-h	147 ± 1 ^a^	179 ± 2 ^b^	83 ± 3 ^b^	80 ± 2 ^b^
CS-oil	114 ± 2 ^b^	158 ± 2 ^c^	75 ± 1 ^c^	73 ± 1 ^c^
CS-oil-h	144 ± 3 ^a^	196 ± 5 ^a^	103 ± 4 ^a^	99 ± 5 ^a^

^1^ Data are presented as mean ± standard deviation, based on three independent analyses. Within a column, means bearing different letters indicate a significant difference at *p* < 0.05. ^2^ CS: native cassava starch, oil: flaxseed oil, h: mild thermal treatment at 65 °C for 7 days. ^3^ Starch pastes (8%, *w*/*w*) prepared using RVA were stored at 4 °C for 24 h and then left at room temperature for 1 h before texture analysis. ^4^ Adhesiveness is expressed as the absolute value of the negative work recorded during the probe withdrawal phase of the TPA cycle.

## Data Availability

The original contributions presented in this study are included in the article. Further inquiries can be directed to the corresponding authors.

## Abbreviations

The following abbreviations are used in this manuscript:

CS	Cassava starch
CLSM	Confocal laser scanning microscopy
FITC	Fluorescein isothiocyanate
DSC	Differential scanning calorimeter
FTIR	Fourier transform infrared spectroscopy
RVA	Rapid viscosity analyzer
EA	Emulsifying ability
ES	Emulsion stability
